# Cancer incidence and mortality rates and trends in Trinidad and Tobago

**DOI:** 10.1186/s12885-018-4625-x

**Published:** 2018-07-04

**Authors:** Wayne A. Warner, Tammy Y. Lee, Kimberly Badal, Tanisha M. Williams, Smriti Bajracharya, Vasavi Sundaram, Nigel A. Bascombe, Ravi Maharaj, Marjorie Lamont-Greene, Allana Roach, Melissa Bondy, Matthew J. Ellis, Timothy R. Rebbeck, Simeon Slovacek, Jingqin Luo, Adetunji T. Toriola, Adana A. M. Llanos

**Affiliations:** 1 Oncology Division, Siteman Cancer Center; Department of Cell Biology and Physiology, Washington University School of Medicine, St. Louis, MO USA; 2 MedSeq HealthCare Solutions, Trincity, Trinidad and Tobago; 3 California State University, Los Angeles, CA USA; 4 Caribbean Cancer Research Initiative, San Fernando, Trinidad and Tobago; 5 Ecology and Evolutionary Biology, University of Connecticut, Storrs, CT USA; 6 Center for Public Health Systems Science, George Warren Brown School of Social Work, Washington University, St. Louis, MO USA; 7 Department of Genetics, Center for Genome Sciences and Systems Biology, Washington University School of Medicine, St. Louis, MO USA; 8 Department of Clinical Surgical Sciences, Faculty of Medical Sciences, University of the West Indies, St. Augustine, Trinidad and Tobago; 9 Dr. Elizabeth Quamina Cancer Registry, Eric Williams Medical Sciences Complex, Mt. Hope, Mt. Hope, Trinidad and Tobago; 10 Department of Educational Services, St. George’s University, St. George’s West Indies, Grenada; 11 Dan L. Duncan Cancer Center, Baylor College of Medicine, Houston, TX USA; 12 Harvard TH Chan School of Public Health and Dana Farber Cancer Institute, Boston, MA USA; 13 Biostatistics Core, Siteman Cancer Center, Washington University School of Medicine, St. Louis, MO USA; 14 Division of Public Health Sciences, Department of Surgery, Washington University School of Medicine, St. Louis, MO USA; 15 Department of Epidemiology, Rutgers School of Public Health and Division of Population Science, Rutgers Cancer Institute of New Jersey, Rutgers University, New Brunswick, NJ USA; 16 Oncology Division, Sections of SCB, BMT, Washington University School of Medicine, Campus Box 8007, 660 S. Euclid Avenue, St. Louis, MO 63110 USA; 17 Department of Epidemiology, Rutgers School of Public Health, 683 Hoes Lane West, Room 211, Piscataway, NJ 08854 USA

**Keywords:** Trinidad and Tobago, Caribbean, Cancer incidence, Cancer mortality, Cancer surveillance, Cancer in populations of African ancestry, Cancer in populations of Indian ancestry

## Abstract

**Background:**

Cancer is the second leading cause of death in the Caribbean, including the islands of Trinidad and Tobago (TT). The population of TT consists of over 1.3 million people with diverse ancestral and sociocultural backgrounds, both of which may influence cancer incidence and mortality. The objective of this study was to examine incidence and mortality patterns and trends in TT.

**Methods:**

Cancer surveillance data on 29,512 incident cancer cases reported to the Dr. Elizabeth Quamina Cancer Registry (population-based cancer registry of TT) between 1995 and 2009 were analyzed. Age-standardized rates, overall and by sex, ancestry, and geography, were reported.

**Results:**

The highest incidence and mortality rates were observed for cancers related to reproductive organs in women, namely, breast, cervical, and uterine cancers, and prostate, lung and colorectal cancers among men. Average incidence rates were highest in areas covered by the Tobago Regional Health Authority (TRHA) (188 per 100,000), while average mortality rates were highest in areas covered by the North West Regional Health Authority (108 per 100,000). Nationals of African ancestry exhibited the highest rates of cancer incidence (243 per 100,000) and mortality (156 per 100,000) compared to their counterparts who were of East Indian (incidence, 125 per 100,000; mortality, 66 per 100,000) or mixed ancestry (incidence, 119 per 100,000; mortality, 66 per 100,000).

**Conclusions:**

Our findings highlight the need for national investment to improve the understanding of the epidemiology of cancer in Trinidad and Tobago, and to ultimately guide much needed cancer prevention and control initiatives in the near future.

**Electronic supplementary material:**

The online version of this article (10.1186/s12885-018-4625-x) contains supplementary material, which is available to authorized users.

## Background

Cancer is the second leading cause of death in the Caribbean and has created tremendous challenges for healthcare services and expenditures throughout the region [1]. The World Health Organization (WHO) projects that cancer incidence will increase by 58%, from 84,703 cases in 2015 to 133,937 cases in 2035, and cancer mortality will increase by 67% during this period, from 52,282 to 87,430 deaths [2]. Aging of the population, improvements in healthcare and economic development has led to a higher prevalence of lifestyle-related risk factors for cancer, including reproductive behaviors, dietary patterns, physical inactivity, obesity, and alcohol and tobacco use. In addition, the prevalence of cancer-associated viral infections (e.g., human papillomavirus, human herpesvirus-8 (HHV8), human T-cell lymphotropic virus-1 (HTLV-1), hepatitis B virus (HBV)), may be higher among Caribbean populations compared to United States (US) populations [3, 4].

In the twin island nation of Trinidad and Tobago (TT), cancer is a leading cause of death much like the rest of the Caribbean [5]. These English-speaking islands are unique in terms of their economy and ancestry. TT, located off the northeastern edge of South America, is one of the richest countries by gross domestic product (GDP) per capita in the Americas and is classified as a high-income economy by the World Bank [6]. This is due to the nation’s industrialized economy, which includes petroleum, natural gas, chemical industries, and food and beverage industries [7]. While TT is classified as a developing country by the International Monetary Fund (IMF) [8] and is a member of the United Nations Conference of Small Island Developing States (SIDS) [9], this nation faces major challenges in its efforts to achieve developed nation status in sectors such as healthcare [10]. TT’s estimated population is 1.4 million [11] with an average life expectancy of 74.61 years [12]. From 1990 to 2010, the demographic profile of TT underwent a transition marked by a declining fertility rate, a decrease in the < 15 years age-group, and a doubling of the > 60 years age-group [5]. While the Trinidad population consists of diverse ancestral groups (including African (31.76%), East Indian (37.01%), Mixed ancestry (23.52%), Chinese, White, and Syrian/Lebanese (< 1%)), as well as religious groups (including Christian, Muslim, and Hindu), the population in Tobago is predominantly of African ancestry (85.29%) and Christian [11, 12]. These demographic patterns have resulted in customs and traditions that have marked the sociocultural development of the islands [13]. Hence, research on the epidemiology and etiology of cancers in TT, in relation to the environment, lifestyle and ancestry, is essential for the success of cancer prevention and control programs and policies.

The literature on the burden of cancer within TT remains relatively barren. Previous studies of cancer in TT have reported site-specific cancer incidence, mortality and survival rates, including for breast, prostate, and gastric cancers [14–19]. However, a comprehensive analysis of cancer incidence and mortality has never been reported. Since 1994, the Dr. Elizabeth Quamina Cancer Registry has served as the National Cancer Registry of TT, using standard cancer registry guidelines and statistical methods [20, 21].

Here, we present on cancer incidence and mortality rates and trends in TT for the overall population, and by sex, geography, ancestry, and age. This is the first epidemiologic examination of cancer rates and trends across all cancer sites in TT for the period 1995 to 2009.

## Methods

We obtained retrospectively collected cancer surveillance data (incidence and mortality) reported between January 1, 1995 and December 31, 2009 to the National Cancer Registry of TT, which represents all of the most current and available data from the cancer registry. The analytic dataset consisted of the 29,512 incident cancers (pediatric and adult cases) reported during the study period. The source of the registry records was previously described [18]. In brief, the registry abstracts data from private and public hospitals across Trinidad and Tobago including all of the main cancer treatment centers. Abstracted data included place of residence, age, sex, ancestry, stage, grade and method of cancer detection. In the registry data file, cancer histology was coded based on WHO *International Classification of Diseases for Oncology (ICD-O)* code C61.9. as supplied by the healthcare institutions [22]. The boundaries for the geographic analysis by corporation and Regional Health Authority (RHA) were previously described [18]. In brief, TT is divided into fifteen governmental administrative corporations and five RHAs for healthcare delivery and administration. Self-identification, medical records, and to a lesser extent, imputation by binary logistic regression were used to determine ancestry [18].

Death certification and population data were obtained from the Trinidad and Tobago Central Statistical Office (CSO) 2000 and 2010 census. The population pyramids for 2000 and 2011 were previously described [11, 12]. Populations estimates for the other study years were calculated through interpolation using the “irregular points of year” estimation method [20, 21]. The CSO collects several population measures including age (single year of age, 5- and 10-year age groups), ethnicity, and sex. From these data points, we calculated the age-standardized incidence and mortality rates (per 100,000 TT population) by age (10-year), sex, geography, ancestry, and individual years based on the 1960 world-standardized population [23, 24]. This methodology was selected for ease of comparison with the International Agency for Research on Cancer (IARC) data which uses the same standardization. The TT case fatality rate was calculated by dividing the number of cancer deaths over the study period by the number of incident cases and then multiplying the resultant ratio by 100 to yield a percentage. For the period 1995–2007, the average time between cancer incidence and cancer death was calculated by taking the average length of the time from the year of incidence to the year of death for the 15,279 cancer deaths recorded during the same time period. For the same time period, the mean survival time (among cancer cases reported in the registry as still living at last contact, *N* = 10,087) was calculated by taking the average of the time from incidence to date of last contact. The geospatial maps were rendered in the R computing environment [25] and Statistical Package of Social Science (SPSS) V.20 (IBM Corporation, Valhalla, NY) was used for analyses.

## Results

### Cancer incidence and mortality rates among men and women

The number and percentage of cancer cases and deaths, along with age-standardized cancer incidence and mortality rates are shown in Table 1. Between 1995 and 2009, there were a total of 29,512 incident primary cancers and 18,216 cancer deaths in TT, with an overall case-fatality rate of 61.7%. For this time period, the average length of time between diagnosis and death was 1 year (range: less than 1 year to 40 years) and the average survival time among living cases was 1.1 year (range: less than 1 year to 14 years). Several basic metrics of data quality from the registry are provided (Additional file 1: Table S1). Of note, the percent of cases registered only on the basis of the death certificate only (DCO) fluctuated from 12.12% in 1995 to 27.63% in 2000 to 10.48% in 2005 and then to 6.32% in 2009. The average over the study period was 18.44%.

**Table 1:** Counts, percentages, and age-standardized incidence (A) and mortality (B) rates per 100,000 for adult and pediatric cancers, Trinidad and Tobago, 1995–2009

**(A) Incidence** Cancer site		Both sexes		Men		Women	
	ICD-10 codes	Count (%)	ASR(W) Rate	Count (%)	ASR(W) Rate	Count (%)	ASR(W) Rate
**Bones & joints**	**C40, C41**	**156 (0.8%)**	**0.8**	**95 (0.6%)**	**0.9**	**61 (0.4%)**	**0.6**
**Brain & other nervous system**	**C70 – C72**	**370 (1.9%)**	**2.0**	**222 (1.5%)**	**2.5**	**148 (1.0%)**	**1.6**
**Breast**	**C50**	**4685 (24.6%)**	**46.6** ^**#**^	**72 (0.5%)**	**0.8**	**4613 (31.6%)**	**46.6**
**Digestive system**	**C15 – C26**	**5967 (31.3%)**	**30.5**	**3340 (22.4%)**	**36.0**	**2627 (18.0%)**	**25.3**
Colon	C18	2023 (10.6%)	10.3	1084 (7.3%)	11.6	939 (6.4%)	9.0
Liver	C22	432 (2.3%)	2.2	242 (1.6%)	2.6	190 (1.3%)	1.8
Pancreas	C25	902 (4.7%)	4.6	489 (3.3%)	5.3	413 (2.8%)	3.9
Rectum	C20	620 (3.3%)	3.2	358 (2.4%)	3.9	262 (1.8%)	2.6
Stomach	C16	999 (5.2%)	5.0	607 (4.1%)	6.5	392 (2.7%)	3.7
Other^a^	C15, 17, 19, 21, 23, 24, 26	991 (5.2%)	4.2	560 (3.7%)	5.0	431 (3.0%)	3.5
**Endocrine system**	**C73 – C75**	**241 (1.3%)**	**1.2**	**73 (0.5%)**	**0.8**	**168 (1.2%)**	**1.6**
**Eye & orbit**	**C69**	**38 (0.2%)**	**0.3**	**25 (0.2%)**	**0.3**	**13 (0.1%)**	**0.2**
**Hematologic** ^b^	**C42, C77**	**1949 (10.2%)**	**10.3**	**1062 (7.1%)**	**11.4**	**887 (6.1%)**	**9.1**
**Oral cavity & pharynx**	**C00 – C14**	**744 (3.9%)**	**3.9**	**532 (3.6%)**	**5.8**	**212 (1.5%)**	**2.1**
**Reproductive system**	**C51 – C63**	**–**	**–**	**6233 (41.7%)**	**65.7**	**4221 (29.0%)**	**42.9**
Penis	C60	–	–	87 (0.6%)	0.8	–	–
Prostate	C61	–	–	6064 (40.6%)	64.0	–	–
Testis	C62	–	–	79 (0.5%)	0.7	–	–
Other male genitals	C63	–	–	3 (0.0%)	0.0	–	–
Cervix uteri	C53	–	–	–	–	1812 (12.4%)	18.1
Corpus uteri	C54	–	–	–	–	1275 (8.7%)	13.4
Ovary	C56	–	–	–	–	885 (6.1%)	8.9
Vagina	C52	–	–	–	–	81 (0.6%)	0.6
Vulva	C51	–	–	–	–	59 (0.4%)	0.6
Other female genitals	C55, C58, C63	–	–	–	–	109 (0.7%)	0.8
**Respiratory system**	**C30 – C34, C37– C39**	**2408 (12.6%)**	**12.5**	**1907 (12.8%)**	**20.7**	**501 (3.4%)**	**4.9**
Larynx	C32	343 (1.8%)	**1.8**	321 (2.1%)	**3.5**	22 (0.2%)	**0.2**
Lung bronchus	C34	1857 (9.7%)	**9.7**	1460 (9.8%)	**15.9**	397 (2.7%)	**3.9**
Other^c^	C30, 31, 33, 37, 38	208 (1.1%)	0.9	126 (0.8%)	1.2	82 (0.6%)	0.7
**Skin (excluding basal & squamous)**	**C44**	**398 (2.1%)**	2.0	**234 (1.6%)**	2.5	**164 (1.1%)**	1.6
**Soft tissue (including heart)**	**C47 – C49**	**274 (1.4%)**	1.4	**151 (1.0%)**	1.6	**123 (0.8%)**	1.3
**Urinary system**	**C64 – C68**	**682 (3.6%)**	**3.6**	**443 (3.0%)**	**4.9**	**239 (1.6%)**	**2.5**
**Ill-defined & unknown**	**C76, C80**	**1146 (6.0%)**	**5.9**	**547 (3.7%)**	**5.9**	**599 (4.1%)**	**5.9**
**TOTAL** ^d^		**19,058**	**98.5**	**14,936**	**159.7**	**14,576**	**146.3**
**(B) Mortality** Cancer site		Overall		Men		Women	
	ICD-10 codes	Count (%)	ASR(W) Rate	Count (%)	ASR(W) Rate	Count (%)	ASR(W) Rate
**Bones & joints**	**C40, C41**	**92 (0.8%)**	**0.5**	**56 (0.6%)**	**0.5**	**36 (0.4%)**	**0.4**
**Brain & other nervous system**	**C70 – C72**	**206 (1.1%)**	**1.1**	**117 (1.2%)**	**1.3**	**89 (1.1%)**	**0.9**
**Breast**	**C50**	**1990 (16.5%)**	**19.2** ^**#**^	**35 (0.4%)**	**0.4**	**1955 (23.5%)**	**19.2**
**Digestive system**	**C15 – C26**	**4459 (36.9%)**	**22.4**	**2502 (25.3%)**	**26.7**	**1957 (23.5%)**	**18.4**
Colon	C18	1331 (11.0%)	**6.6**	719 (7.3%)	**7.6**	612 (7.4%)	**5.7**
Liver	C22	400 (3.3%)	**2.0**	223 (2.3%)	**2.4**	177 (2.1%)	**1.6**
Pancreas	C25	796 (6.6%)	**4.0**	436 (4.4%)	**4.7**	360 (4.3%)	**3.4**
Rectum	C20	338 (2.8%)	1.7	199 (2.0%)	2.1	139 (1.7%)	1.3
Stomach	C16	841 (7.0%)	4.2	503 (5.1%)	5.3	338 (4.1%)	3.2
Other^a^	C15, 17, 19, 21, 23, 24, 26	753 (6.2%)	3.8	422 (4.3%)	4.5	331 (4.0%)	3.2
**Endocrine system**	**C73 – C75**	**125 (1.0%)**	0.6	**42 (0.4%)**	0.5	**83 (1.0%)**	0.8
**Eye & orbit**	**C69**	**11 (0.1%)**	0.1	**6 (0.1%)**	0.1	**5 (0.1%)**	0.1
**Hematologic** ^b^	**C42, C77**	**1407 (11.6%)**	7.3	**769 (7.8%)**	8.2	**638 (7.7%)**	6.5
**Oral cavity & pharynx**	**C00 – C14**	**446 (3.7%)**	2.3	**330 (3.3%)**	3.6	**116 (1.4%)**	1.2
**Reproductive system**	**C51 – C63**	**–**	**–**	**3772 (38.1%)**	**38.2**	**2356 (28.3%)**	**23.5**
Penis	C60	–	–	37 (0.4%)	0.4	–	–
Prostate	C61	–	–	3704 (37.4%)	37.5	–	–
Testis	C62	–	–	29 (0.3%)	0.3	–	–
Other male genitals	C63	–	–	2 (0.0%)	0.0	–	–
Cervix uteri	C53	–	–	–	–	974 (11.7%)	9.7
Corpus uteri	C54	–	–	–	–	632 (7.6%)	6.4
Ovary	C56	–	–	–	–	582 (7.0%)	5.8
Vagina	C52	–	–	–	–	42 (0.5%)	0.4
Vulva	C51	–	–	–	–	38 (0.5%)	0.4
Other female genitals	C55, C58, C63	–	–	–	–	87 (1.0%)	0.8
**Respiratory system**	**C30 – C34, C37– C39**	**1777 (14.7%)**	**9.2**	**1409 (14.2%)**	**15.2**	**368 (4.4%)**	**3.6**
Larynx	C32	184 (1.5%)	0.9	174 (1.8%)	1.8	10 (0.1%)	0.1
Lung bronchus	C34	1477 (12.2%)	7.7	1162 (11.7%)	12.7	315 (3.8%)	3.1
Other^c^	C30, 31, 33, 37, 38	116 (1.0%)	0.6	73 (0.7%)	0.7	43 (0.5%)	0.4
**Skin (excluding basal & squamous)**	**C44**	**141 (1.2%)**	**0.7**	**83 (0.8%)**	**0.9**	**58 (0.7%)**	**0.5**
**Soft tissue (including heart)**	**C47 – C49**	**140 (1.2%)**	**0.8**	**83 (0.8%)**	**0.9**	**57 (0.7%)**	**0.6**
**Urinary system**	**C64 – C68**	**419 (3.5%)**	2.1	**276 (2.8%)**	3.0	**143 (1.7%)**	1.4
**Ill defined & unknown**	**C76, C80**	**875 (7.2%)**	4.4	**416 (4.2%)**	4.4	**459 (5.5%)**	4.4
**TOTAL** ^d^		**12,088**	**61.5**	**9896**	**103.8**	**8320**	**81.5**

NOTE: Percentages may not sum to 100% due to rounding. Information refering to major organ systems are bolded

^a^Anus, anal canal, bile tract, esophagus, gall bladder, gastrointestinal, rectosigmoid junction, small intestine

^b^Myeloma, lymphoma, leukemia

^c^Accessory sinus, ear/nose, heart, pleura, respiratory tract, thymus, trachea

^d^Excludes genital cancer

**Fig. 1:**
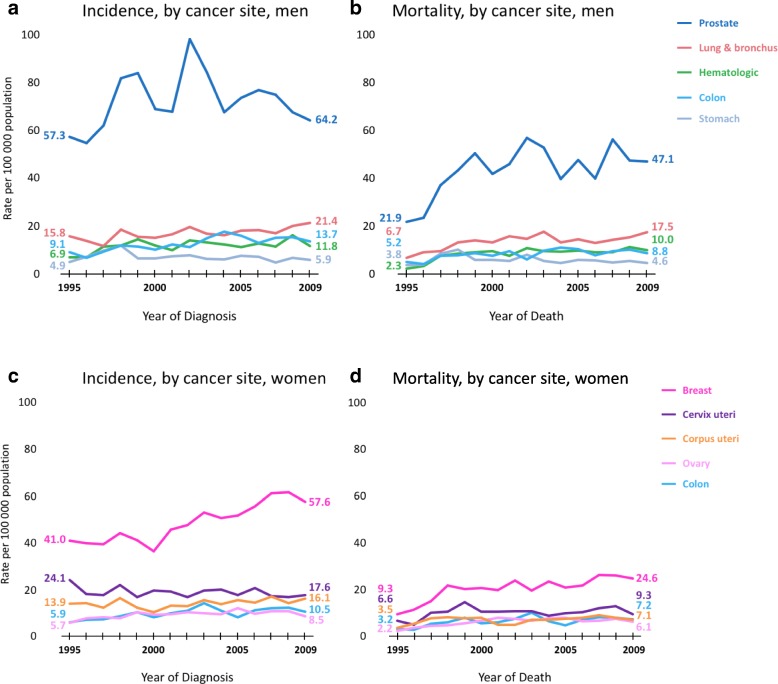
Trends in rates for selected cancers by sex, Trinidad and Tobago, 1995 to 2009. Rates are age adjusted to the 1960 world standard population. **a, b** incidence and mortality cancer rates for men and (**c, d**) incidence and mortality cancer rates for women

Overall, cancer incidence and mortality rates were 13.4 and 22.3% higher, respectively, among men than women. Cancer incidence and mortality trends by sex in TT are shown in Fig. 1. The overall age-standardized cancer incidence rate among TT men was 159.7 per 100,000, while the overall mortality rate was 103.8 per 100,000. The most commonly diagnosed cancers among men were prostate (64.0 per 100,000), lung and bronchus (15.9 per 100,000), colon (11.6 per 100,000), hematologic (11.4 per 100,000) and stomach (6.5 per 100,000). Similarly, the cancers with the highest mortality rates among men were prostate (37.5 per 100,000), lung and bronchus (12.7 per 100,000), hematologic (8.2 per 100,000), colon (7.6 per 100,000) and stomach cancer (5.3 per 100,000). Among TT women, the overall age-standardized cancer incidence rate was 146.3 per 100,000, while the overall mortality rate was 81.5 per 100,000. The most commonly diagnosed cancers among women were breast (46.6 per 100,000), cervix uteri (18.1 per 100,000), corpus uteri (13.4 per 100,000), hematologic (9.1 per 100,000), colon (9.0 per 100,000) and ovarian cancer (8.9 per 100,000). The cancers with the highest mortality were breast (18.4 per 100,000), cervix uteri (9.7 per 100,000), corpus uteri (6.4 per 100,000), hematologic (6.5 per 100,000) and ovarian (5.8 per 100,000) (Table 1A, B).

### Cancer incidence and mortality rates by geography

**Fig. 2:**
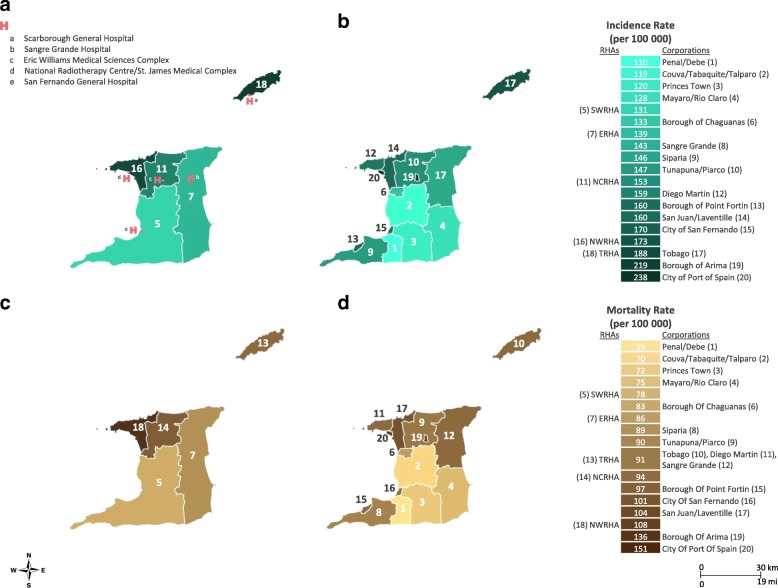
Geospatial maps of cancer incidence and mortality rates in Trinidad and Tobago 1995–2009: (Top panel, left to right) Age-standardized incidence rates for all (**a**) Regional Health Authorities and (**b**) Corporations, and age-standardized mortality rates for all (**c**) Regional Health Authorities and (**d**) Corporations. Rates are age adjusted to the 1960 world standard population. H, Hospital

The geographical area of residence corresponded with Ministry of Health RHAs (Fig. 2). There are five RHAs in TT – North West Regional Health Authority (NWRHA); North Central Regional Health Authority (NCRHA); South West Regional Health Authority (SWRHA); Eastern Regional Health Authority (ERHA); and Tobago Regional Health Authority (TRHA) – responsible for direct provision of healthcare services in their respective catchment area [18]. As shown in Fig. 2, age-standardized cancer incidence and mortality rates varied by RHA. Average incidence rates were highest in areas covered by the TRHA (188 per 100,000), followed by NWRHA (173 per 100,000), and NCRHA (153 per 100,000), compared to ERHA (139 per 100,000) and SWRHA (131 per 100,000). Overall, average mortality rates were highest in areas covered by NWHRA (108 per 100,000), followed by NCRHA and TRHA, (94 and 91 per 100,000, respectively). The NWRHA covers the area that includes the capital city of Port-of-Spain, which had the highest overall age-standardized cancer incidence (238 per 100,000) and mortality (151 per 100,000). Corporations within the SWRHA catchment area, Penal and Debe (110 per 100,000) and Couva, Tabaquite, and Talparo (119 per 100,000) had among the lowest overall cancer incidence rates. Penal and Debe had the lowest cancer mortality (59 per 100,000).

### Cancer incidence and mortality by ancestry

**Fig. 3:**
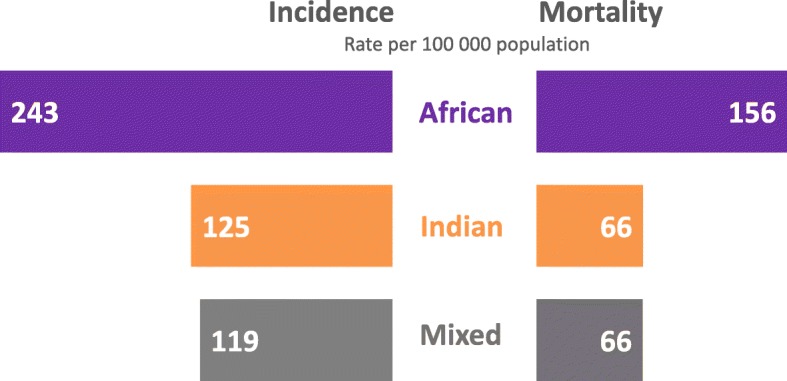
Overall cancer incidence and mortality rates in Trinidad and Tobago by ancestry, 1995–2009. Rates are age adjusted to the 1960 world standard population

**Fig. 4:**
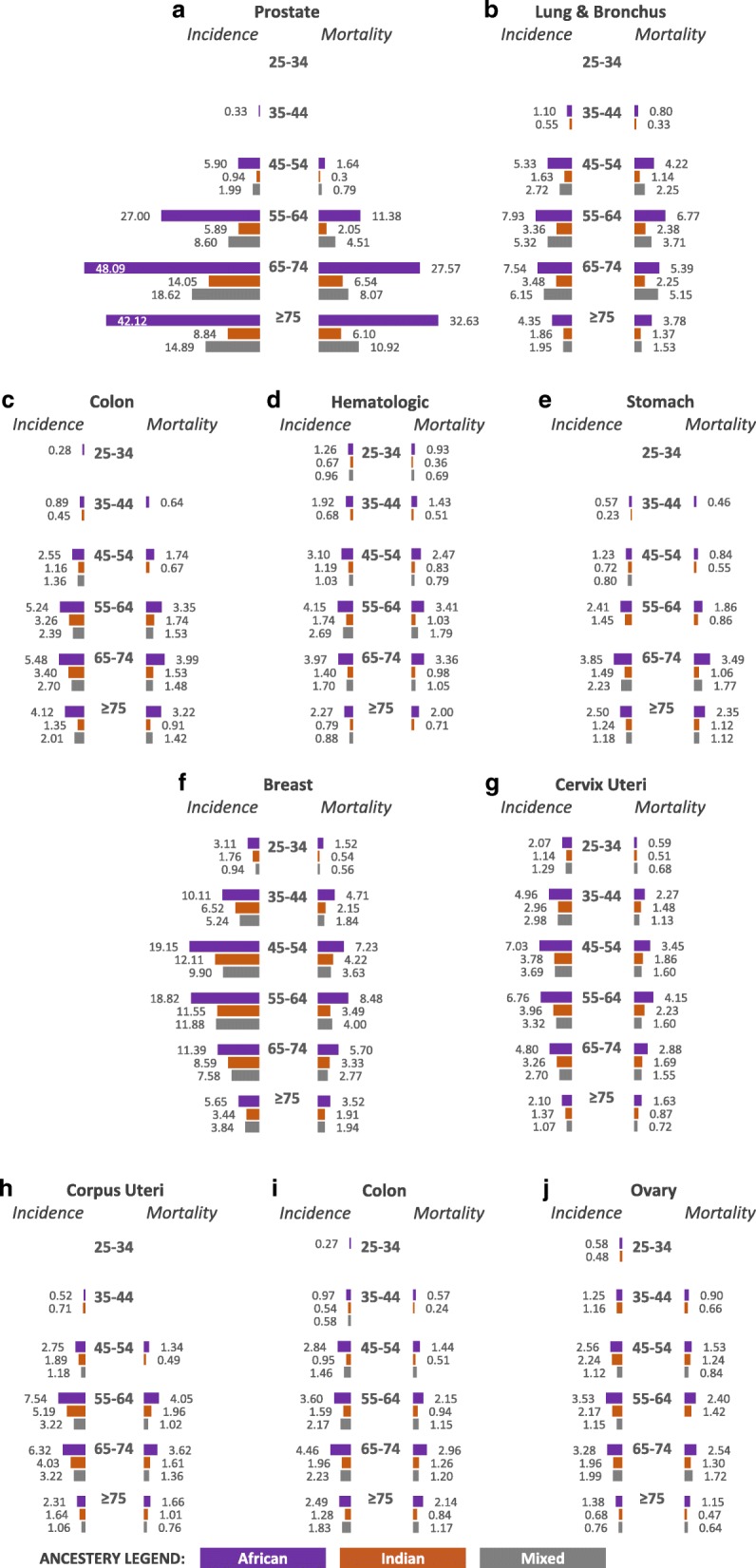
Age-standardized incidence and mortality rates for the leading cancer sites by sex (**a-e**, male; **f-j**, female), ancestry (purple, African ancestry; orange, Indian ancestry; gray, Mixed ancestry), and age groups, Trinidad and Tobago, 1995–2009. All bars are uniformly scaled. Data for persons under 24 are presented in Additional file 2: Table S2

The highest cancer incidence (243 per 100,000) and mortality (156 per 100,000) rates were observed among individuals of African ancestry compared to Indian or mixed ancestry (Fig. 3). Cancer incidence and mortality rates by sex, ancestry, and age in TT are shown in Fig. 4**.** The highest burden of cancer for both men and women were observed among those ≥45 years. Among TT men, cancers with the highest incidence and mortality (prostate, colon, hematologic and stomach cancers) were observed among those 65–74 years (Fig. 4a-e). However, the highest lung cancer rates were observed among those aged 55–64 years (Fig. 4b). Among TT women, the highest breast and cervical cancer incidence and mortality rates were observed among those 45–54 years (Fig. 4f-j). Women 55–64 years experienced the highest corpus uteri incidence, while colon cancer and ovarian cancer occurred most frequently among women 65–74 years (Fig. 4h-j). The highest mortality rates for corpus uteri cancer were observed among women 55–64 years, while the highest mortality rates for colon and ovarian cancers were observed for women 65–74 years (Fig. 4h-j). Incidence and mortality data for those under 24 is provided (Additional file 2: Table S2).

### Stage distribution of selected cancers

**Fig. 5:**
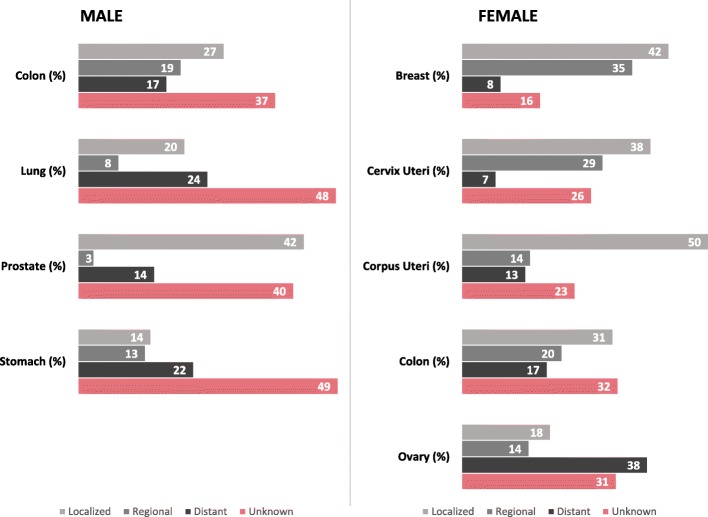
Stage distribution of selected cancers by sex, Trinidad and Tobago, 1995–2009. Stage categories may not sum to 100% because of rounding

Figure 5 shows the distribution of stage at diagnosis among the leading cancers by sex and ancestry. Most prostate cancers were diagnosed at localized stage (African 42%, Indian 46%, and mixed ancestry 39%). More than 15% of lung and bronchus (African 21%, Indian 19%, and mixed ancestry 33%) and stomach cancers (African 18%, Indian 19%, and mixed ancestry 28%) occurred in the distant stage. Less than 15% of breast (African 10%, Indian 6%, and mixed ancestry 8%) and cervix uteri cancers (African 10%, Indian 6%, and mixed ancestry 8%) occurred in the distant stage. More than 30% of all ovarian cancers (African 36%, Indian 27%, and mixed ancestry 31%) occurred in the distant stage. Strikingly, across all sites, there was a high percentage (12–57%) of cancers with unknown stage.

## Discussion

This is the first epidemiologic study to examine TT cancer rates and trends across all cancer sites, by age, ancestry, geography and sex, with a focus on the cancers with highest incidence rates. We found that incident cases of prostate, lung, colon, stomach, and hematologic cancers were most common among men, while among women, the cancers with the highest incidence were breast, cervical, endometrial, colon, and ovarian. Overall, the incidence rates were highest in Tobago and the area covered by the NWRHA (which contains the capital city of Port-of-Spain), while cancer mortality was highest in NWRHA. The highest incidence and mortality rates were observed among adults aged ≥45 years. Except cervical and breast cancers, most cancers had a significant proportion of cases (> 10%) diagnosed at distant stage and all cancers except breast had > 20% with unknown stage. TT nationals of African ancestry had the highest incidence and mortality rates. The high overall case-fatality rate reflects the need for improved strategies to reduce cancer mortality in TT.

Evidence suggests that many of the most prevalent cancers among men and women in TT are likely attributable to preventable lifestyle factors (e.g., associated with the “westernized lifestyle” of developed countries). Tobacco use, obesity, pathogens, physical inactivity, diet, and alcohol are among the known lifestyle factors associated with increased cancer incidence [26–29]. A recent study in TT reported that the current prevalence of tobacco use among men (33.5%) is significantly higher than among women (9.4%), which may contribute to the higher rate of lung cancer among men [30]. Additionally, the study found that the overall mean body mass index (BMI) for women and men in TT was 27.4 kg/m^2^ and 25.6 kg/m^2^, respectively, and that > 55.7% of the population was overweight or obese (BMI ≥25) [30]. A recent IARC working group comprehensive review of multiple datasets, concluded that excess body fat causes cancer in multiple anatomical sites, including those identified in this study as placing the highest burden in TT [31, 32]. Numerous studies have reported the causal relationship between physical inactivity and cancer [33–35], and while the amount of total physical activity (PA) required to lower the risk of specific cancers in a dose-response fashion has not been established, the WHO recommends at least 600 metabolic equivalents of task (MET) minutes/week for health benefits [36]. Strikingly, a recent study in TT reported that the median PA approximated 300.30 (MET minutes/week) (median, 149.1 MET minutes/week) [30]. Focused cancer prevention initiatives in TT targeting obesity reduction and an increase in PA might have value in reducing the risk of cancer.

The TT population of African ancestry suffers a higher cancer burden across all cancer sites. This is similar to studies reporting that members of the African diaspora suffer a disproportionate cancer burden compared to other groups [37, 38]. Interestingly, we found that in Tobago, with its relatively homogeneous African ancestry population, there was a higher overall cancer incidence rate (187.7 per 100,000), driven primarily by the incidence rates of breast and prostate cancers. Surprisingly, while the incidence rates are high, mortality rates are relatively low (91.3 per 100,000). Further exploration is needed to evaluate the causes of the excess cancer burden, which may include genetic variation, tumor biology, and additional factors that have been understudied in the TT population. The contribution of tumor biology, genomics, comorbidities and patterns of care to the higher cancer burden and the observed disparities is not clear and warrant further exploration.

There are no national cancer screening programs in TT. However, the results from a population-based prostate cancer screening in Tobago between September 1997 and June 2001 are particularly worth noting. Here screening for prostate cancer using serum prostate-specific antigen (PSA) and digital rectal exam (DRE) revealed a very high prevalence of clinically-detected prostate cancer [39]. While it is true that increased prostate cancer screening leads to higher incidence, data support the hypothesis that populations of African descent share genetic and/or lifestyle factors that underlie an elevated prostate cancer risk [40, 41]. A recent study compared the effect of birthplace on prostate cancer risk comparing US-born men and men from two Caribbean countries (Guyana and TT) [19]. This study found that Caribbean-born men were diagnosed at an older age and had worse 5-year survival than US-born men, although among Caribbean-born men who immigrated to the US, 5-year survival was similar to that of US-born African American men [19]. A similar study of breast cancer reported lower survival among Caribbean women of African ancestry living in the Caribbean compared to those who migrated to the US, compared to US-born African American women [15]. This gap could be due to the intersection of screening, health literacy, tumor biology, genomics, and patterns of care issues [42].

This study has certain limitations, perhaps the greatest of which was the limited quality of cancer surveillance data currently available on the TT population. For example, large proportions of data on cancer stage were missing and molecular subtype for breast cancer, for example was not reported, which precludes interpretation of some of the study’s findings. There are issues related to the data validity that can be addressed by an increase in the quality of the data collected and improved steps to have its data included in Cancer Incidence in Five Continents. While the average DCO cases of 18.44% falls below the threshold set by IARC for inclusion in Cancer Incidence in Five Continents [43], it still reflects a need for the TT cancer registry to improve data validity. Fluctuations in data quality over the study period might have impacted some of the trends we report. This further highlights the need to strengthen the capacity of the registry. Another limitation was the inability to access and examine cancer screening data, which would be an important consideration in terms of the mortality disparities reported herein. It is plausible that disparities in cancer mortality were associated with disparities in access and utilization of cancer screening tests, because of differences in socioeconomic status, geography and/or other factors. Furthermore, under the equal access to care model in TT, initiation and receipt of optimal cancer treatment, as well as cancer care overall, may be dependent upon a patient’s place of residence, and therefore related to differences in resource allocation by RHAs. Another limitation was the lack of information on where patients sought cancer care (i.e., within the catchment area of their assigned RHA or elsewhere). Cancer surveillance data collected in TT is not routinely linked to tumor clinicopathological data leading to compromised data accuracy, utility and quality. Additionally, TT does not have electronic health records, further limiting the availability for this detailed cancer data. Similar to National Cancer Institute’s Surveillance, Epidemiology and End Results (SEER) registries, data on behavioral characteristics of cancer cases are not included in the TT cancer registry. This highlights the need for improved cancer surveillance that can accurately inform and support cancer prevention and control initiatives [44]. Despite these limitations, this study highlights the need for national strategic investment in cancer epidemiology, prevention and control.

## Conclusion

In conclusion, the findings of this study demonstrate that in TT, the highest incidence and mortality rates were observed for cancers related to reproductive organs in women, and prostate, lung and colorectal cancers among men, with differences observed by geography and ancestry. In developed countries, prostate and breast cancer rates are decreasing, unlike in TT, where the rates are increasing. Thus, the findings reported herein highlight the need for national investment to improve the understanding of the epidemiology of cancer in Trinidad and Tobago, and to ultimately guide much needed cancer prevention and control initiatives in the near future. Cancer prevention efforts should be strategically increased, particularly among those cancers that are attributable to lifestyle choices. The high proportion of cancers diagnosed at distant and unknown stages, also highlights the need for improvements in cancer screening and treatment initiatives in TT. Considering the high burden of cancer in TT, we expect that findings from this study will inform future policies, particularly related to resource allocation across the cancer care continuum in TT. Additionally, it is clear that capacity-building within the cancer registry (e.g., to mandate standardized data collection and routine molecular subtyping of tumors) is essential for improved cancer surveillance. This will undoubtedly improve the quality of data available for future research and will play an instrumental role in improving cancer care in TT.

## Additional files

Additional file 1: **Table S1.** Basis of diagnosis for all cancer cases recorded in the National Cancer Registry of TT, 1995–2009. (DOCX 14 kb)

Additional file 2: **Table S2.** Age-standardized incidence and mortality rates for two of the ten leading cancer sites by sex, and ancestry, for persons less than 24 years old, Trinidad and Tobago, 1995–2009. (DOCX 15 kb)

## Abbreviations

BMI: Body Mass Index
CSO: Central Statistical Office
DCO: Death Certificate Only
DRE: Digital Rectal Exam
ERHA: Eastern Regional Health Authority
GDP: Gross Domestic Product
HBV: Hepatitis B Virus
HHV8: Human Herpesvirus-8
HTLV-1: Human T-Cell Lymphotropic Virus-1
IARC: International Agency for Research on Cancer
ICD-O: International Classification of Diseases for Oncology
IMF: International Monetary Fund
MET: Metabolic Equivalents of Task
NCRHA: North Central Regional Health Authority
NWRHA: North West Regional Health Authority
PA: Physical Activity
PSA: Prostate-Specific Antigen
RHA: Regional Health Authority
SEER: Surveillance, Epidemiology and End Results
SIDS: United Nations Conference of Small Island Developing States
SPSS: Statistical Package of Social Science
SWRHA: South West Regional Health Authority
TRHA: Tobago Regional Health Authority
TT: Trinidad and Tobago
US: United States
WHO: World Health Organization
