# Supplementary material for: Cancer incidence and mortality rates and trends in Trinidad and Tobago
Source: BMC Cancer. 2018 Jul 4;18:712. doi: 10.1186/s12885-018-4625-x (PMC6032795; doi:10.1186/s12885-018-4625-x)
Supplement: Supplementary file 1 — Table S1. Basis of diagnosis for all cancer cases recorded in the National Cancer Registry of TT, 1995–2009. (DOCX 14 kb) [file 12885_2018_4625_MOESM1_ESM.docx]

| **Table S1.** Basis of diagnosis for all cancer cases recorded in the National Cancer Registry of TT, 1995-2009. | |
| --- | --- |
| **Method** | **Percent of cases, (%)** |
| Laboratory | 0.16 |
| Histology of metastases | 0.27 |
| Cytology | 0.64 |
| Surgery/Autopsy | 0.70 |
| Unknown | 0.80 |
| Clinical only | 0.97 |
| Clinical investigation/Ultrasound | 3.06 |
| Autopsy/Histology | 4.89 |
| Death certificate only | 18.44 |
| Histology of primary | 70.07 |
