# Supplementary material for: Cancer incidence and mortality rates and trends in Trinidad and Tobago
Source: BMC Cancer. 2018 Jul 4;18:712. doi: 10.1186/s12885-018-4625-x (PMC6032795; doi:10.1186/s12885-018-4625-x)
Supplement: Supplementary file 2 — Table S2. Age-standardized incidence and mortality rates for two of the ten leading cancer sites by sex, and ancestry, for persons less than 24 years old, Trinidad and Tobago, 1995–2009. (DOCX 15 kb) [file 12885_2018_4625_MOESM2_ESM.docx]

| **Table S2.** Age-standardized incidence and mortality rates for two of the ten leading cancer sites by sex, and ancestry, for persons less than 24 years old, Trinidad and Tobago, 1995-2009. | | | | | | |
| --- | --- | --- | --- | --- | --- | --- |
|  |  | ***Among Men*** | |  | ***Among Women*** | |
| **Age group** | **Ancestry** | **Hematologic Incidence** | **Hematologic Mortality** |  | **Ovary Incidence** | **Ovary Mortality** |
| **≤14** | African | 1.12 | 0.46 |  | 0 | 0 |
|  | Indian | 2.32 | 0.74 |  | 0 | 0 |
|  | Mixed | 1.08 | 0.77 |  | 0 | 0 |
|  |  |  |  |  |  |  |
| **15-24** | African | 0.82 | 0.58 |  | 0.31 | 0 |
|  | Indian | 1.14 | 0.54 |  | 0.35 | 0 |
|  | Mixed | 0.51 | 0 |  | 0 | 0 |

NOTE: Data for the remaining sites (men- prostate, lung and bronchus, colon, and stomach; women- breast, cervix uteri, corpus uteri, and colon) were not shown as the rates were zero.
